# Mechanism and performance of lithium–oxygen batteries – a perspective

**DOI:** 10.1039/c7sc02519j

**Published:** 2017-07-31

**Authors:** Nika Mahne, Olivier Fontaine, Musthafa Ottakam Thotiyl, Martin Wilkening, Stefan A. Freunberger

**Affiliations:** a Institute for Chemistry and Technology of Materials , Graz University of Technology , Stremayrgasse 9 , 8010 Graz , Austria . Email: freunberger@tugraz.at; b Institut Charles Gerhardt Montpellier , UMR 5253, CC 1701 , Université Montpellier , Place Eugène Bataillon , 34095 Montpellier Cedex 5 , France; c Réseau sur le Stockage Electrochimique de l'énergie (RS2E) , FR CNRS , France; d Department of Chemistry , Indian Institute of Science Education and Research (IISER) , Dr Homi Bhabha Road, Pashan , Pune , 411008 , India

## Abstract

Rechargeable Li–O_2_ batteries have amongst the highest formal energy and could store significantly more energy than other rechargeable batteries in practice if at least a large part of their promise could be realized. Realization, however, still faces many challenges than can only be overcome by fundamental understanding of the processes taking place. Here, we review recent advances in understanding the chemistry of the Li–O_2_ cathode and provide a perspective on dominant research needs. We put particular emphasis on issues that are often grossly misunderstood: realistic performance metrics and their reporting as well as identifying reversibility and quantitative measures to do so. Parasitic reactions are the prime obstacle for reversible cell operation and have recently been identified to be predominantly caused by singlet oxygen and not by reduced oxygen species as thought before. We discuss the far reaching implications of this finding on electrolyte and cathode stability, electrocatalysis, and future research needs.

## Abstract 2

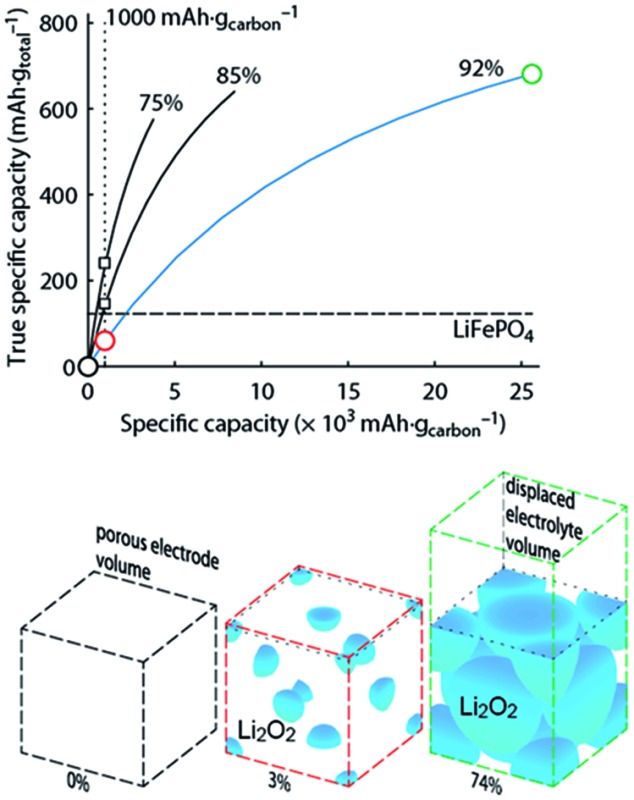
Rechargeable Li–O_2_ batteries have amongst the highest formal energy and could store significantly more energy than other rechargeable batteries in practice if at least a large part of their promise could be realized.

## 1. Introduction

Raising energy storage beyond the limits of current battery technology has become a societal demand and focus of much Frontier research. The achievable limits of Li-ion batteries with respect to energy, material sustainability and cost will likely not satisfy the needs. This motivates ambitious approaches with ‘beyond-intercalation chemistries’.^1–3^ They store charge instead of intercalation by fundamentally different reactions. These include replacing the graphite anode by Li metal and intercalation cathodes by the O_2_ cathode to form the Li–O_2_ (air) battery, which is considered the battery with the highest specific energy. The O_2_ cathode comprises a porous, electrolyte filled electron conducting matrix, wherein O_2_ from the ambiance is reduced on discharge to form Li_2_O_2_ and the reverse process on charge.

There is lots of ambiguity of what energy a Li–O_2_ cell could store, despite it being the motivation for the research. The problem arises from confusing formal capacity (1168 mA h g^–1^, 2500 mA h cm^–3^ Li_2_O_2_) with theoretical capacity (Li_2_O_2_ including the minimum electron and ion conductor to allow the storage process O_2_ + 2e^–^ + 2Li^+^ ↔ Li_2_O_2_ to take place) and achieved true capacity (Li_2_O_2_ including the used electron and ion conductor).^4^ We thus shall first discuss realistic performance metrics.

The Li–O_2_ battery combines two challenging electrodes. In most cases a Li metal anode is used, which is, despite decades of research, still associated with poor coulombic efficiencies.^3^ Other high capacity anodes such as Si may also be considered but likely require in either case protection against O_2_ ingress from the cathode. Until a couple of years ago there was hardly any knowledge on the O_2_/Li_2_O_2_ redox couple in aprotic media. Reactive species involved in the cycling mechanism, which challenge the stability of electrolyte and electrode material, turned out to be another critical research direction. Next to these scientific and materials challenges practical realization further faces engineering challenges with cell construction and air handling. Only understanding the science may thus form the foundation for tackling the engineering.

In this perspective we focus on the science underpinning the O_2_ cathode. After dealing with performance we discuss the current understanding of Li_2_O_2_ formation and decomposition on cycling, followed by measures of reversibility, mechanisms that degrade electrolyte and electrode components and porous cathode design. Potentially transformative ideas start with much enthusiasm and hyped expectations. Thereafter, illusions of low hanging fruit fall and only going the extra mile for true understanding can allow progress to continue. The field of Li–O_2_ batteries is now in the latter stage. Real progress has been achieved with mechanistic understanding in the last years, which now puts us in a better position than ever to state that the disillusioned view of Li–O_2_ never leaving the state of a cell with low capacity, rate, energy efficiency and cycle life is too pessimistic. Yet it is unclear whether it can eventually lead to a technology.

## 2. True performance metrics – myths, reality, and reporting standards

When performance is the argument for research work then data need to stand up to it. An alleged 5 to 10 fold theoretical higher specific energy in comparison to current LIBs is often found as justification when papers on the topic are introduced. However, these numbers stem from very simplistic views and are not realistic even in theory.^4,5^

Departing from the intercalation concept of LIBs does generally not allow for a stable framework in the active material. In the Li–O_2_ cathode this means that the full volume of Li_2_O_2_ forms/disappears during discharge/charge. The basic charge storage process at the cathode is linking the redox moiety O_2_ to electron and ion transport according to O_2_ + 2e^–^ + 2Li^+^ ↔ Li_2_O_2_. However, Li_2_O_2_ can, akin to most storage materials, not be cycled anywhere near to the bulk substance. Ion and electron transport are too poor to allow for practical bulk material electrodes. To provide simultaneous contact with ionic pathways to the electrolyte and electronic pathways to the current collector an electrolyte filled porous cathode is used. The capacity at a given initial porosity is determined by the degree of pore filling. Thus, beyond the scale of the single Li_2_O_2_ particle, the electrode including electrolyte becomes the actual Li^+^ host structure, which is required to fulfil the charge storage processes of linking formal ion host particles (Li_2_O_2_) to electron and ion transport. This introduces a ‘super-host structure’ that becomes an indispensable and integral part of the cell chemistry in a given electrode architecture and needs to be accounted for when reporting performance.

**Fig. 1:**
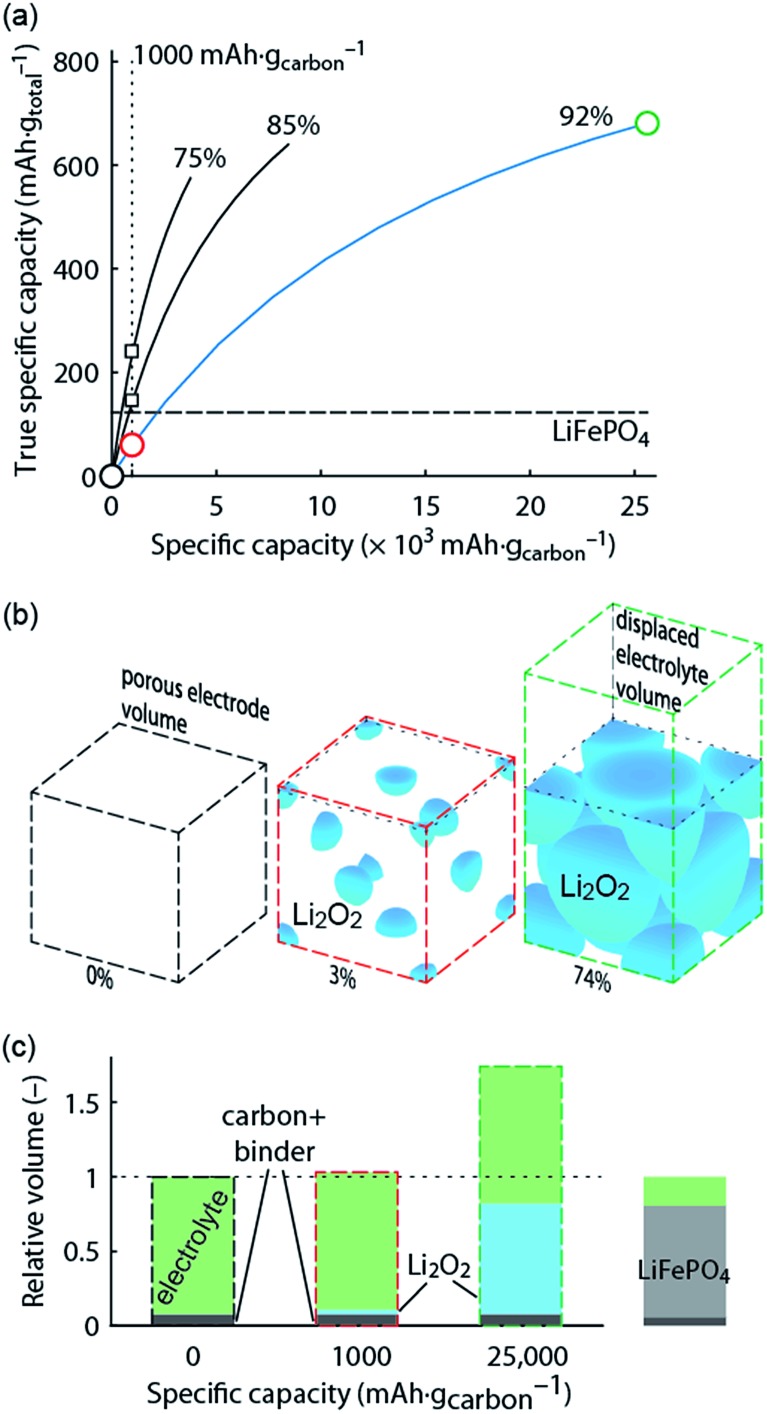
True electrode capacity with limited capacity cycling. (a) True capacity of a Li–O_2_ cathode as a function cycling capacity per mass of carbon substrate for three cases of initial porosity (percentages above the curves). 4% volume are accounted for the binder, and the carbon volume fraction is adapted to yield the initial porosity. At an initial porosity of 92% a Li_2_O_2_ volume occupation of 74% (fcc packing) corresponds to 80% filling of the available pore space. The same 80% filling of the available pore space is assumed for the other initial porosities. The analogous value for the intercalation material LiFePO_4_ is shown for comparison. (b) Space filling of spherical Li_2_O_2_ inside the porous electrode volume with fixed sphere centres and the displaced electrolyte volume (together the super-host structure) at 0, 1000, and 25 000 mA h g_carbon_^–1^, respectively (indicated by the circles in a). Sphere sizes are to scale and the numbers indicate their volume occupation in the porous electrode volume. (c) Volumes of the electrode components at these capacities normalized to the full electrode volume in the delithiated state. Values for a LiFePO_4_ cathode are shown for comparison and demonstrate a very different electrolyte/active material ratio. The figure is adapted from ref. 4 with permission of NPG.

What capacity could the Li–O_2_ cathode with a reasonable super-host structure at best achieve and how does it compare to intercalation chemistries? One can assume that the Li_2_O_2_ particles could at best be packed with 74% volume occupation into a face centred cubic (fcc) structure, the theoretically limiting case. When charged the porous electrode is filled by electrolyte, which is displaced upon Li_2_O_2_ growth. Fig. 1 illustrates the relationship between formal host material and the super-host structure, and the maximum true specific capacity, which reaches ∼700 mA h g_total_^–1^, which is higher than intercalation electrodes.

Metal–O_2_ batteries are special in that the positive electrode does not contain the redox material in the charged state that could be taken as a reference for capacity. Thus, it is convenient to report capacities per weight of porous electron conductor. Often, up to several 10 000 mA h g_carbon_^–1^ are reached as first discharge capacities, which compare superficially favourably with some 100 mA h g^–1^ for intercalation materials.^3,6,7^ As a result of difficulties to cycle at full capacity it has become a habit to report cycling at, *e.g.*, 1000 mA h g_carbon_^–1^, which may still seem a lot in comparison to LIBs. Formal capacities are, however, easily misjudged since true capacities strongly depend on initial porosity and thus the substrate/electrolyte ratio as indicated by the vertical dotted line in Fig. 1a; true capacity at 1000 mA h g_carbon_^–1^ grows with decreasing initial porosity due to the growing electrolyte/Li_2_O_2_ ratio at shallow discharge, Fig. 1b and c.

Limited-capacity cycling often allows simulating a large possible cycle number even if the same cell at full discharge would not reach more than a few cycles and cumulative capacity equating to only a few full cycles. Clearly, overly capacity-limited cycling is not suitable to demonstrate large reversible capacities for many cycles. Yet, it is a common feature of beyond-intercalation chemistries that reasonably capacity-limited cycling can be enabling for cyclability and at the same time yield significant improvement over intercalation if the capacity on a total weight basis is kept in mind as shown in Fig. 1a.^4^

For higher true capacities than intercalation electrodes it is crucial to achieve an as high as possible packing density of Li_2_O_2_ and to minimize inactive mass and volume. Low packing density and overly restricted depth-of-cycling likely result in no advantage over intercalation electrodes as demonstrated in Fig. 1. Reporting capacity with respect to the porous substrate mass does not reveal whether the electrode performs better than an intercalation electrode. A fair assessment requires therefore giving capacity per total electrode mass and volume. Unfortunately, many studies do not report the required measures to work out full electrode performance metrics. The following parameters are required to do so with electrode thickness and electrolyte loading being the only parameters beyond typically reported ones.

(1) The thickness of the porous electrode.

(2) The mass fractions of all components (carbon, binder, and electrolyte) as obtained from the mass fractions of all solids, their loading and the loading of electrolyte per unit electrode area.

(3) The volume fractions of all electrode components are then obtained from the mass fractions and the densities.

(4) With these measures it is straightforward to convert the capacity with respect to substrate into true capacity per mass and volume of total electrode including electrolyte.

These parameters are easily obtained and journal editors and referees are urged to insist on them being provided for papers. There is no theoretical barrier for the Li–O_2_ cathode to achieve higher true capacity than intercalation cathodes also in practice; key is high active material packing density and a small inactive/active material ratio.

## 3. Reaction mechanism at the Li–O_2_ cathode

With true energy depending crucially on filling of the available pore space, the mechanism by which Li_2_O_2_ is forming/decomposing attains paramount importance. It further directly impacts the stability of the cell components and rechargeability *via* the reactivity of the intermediates.

### 3.1. Li_2_O_2_ formation on discharge

**Fig. 2:**
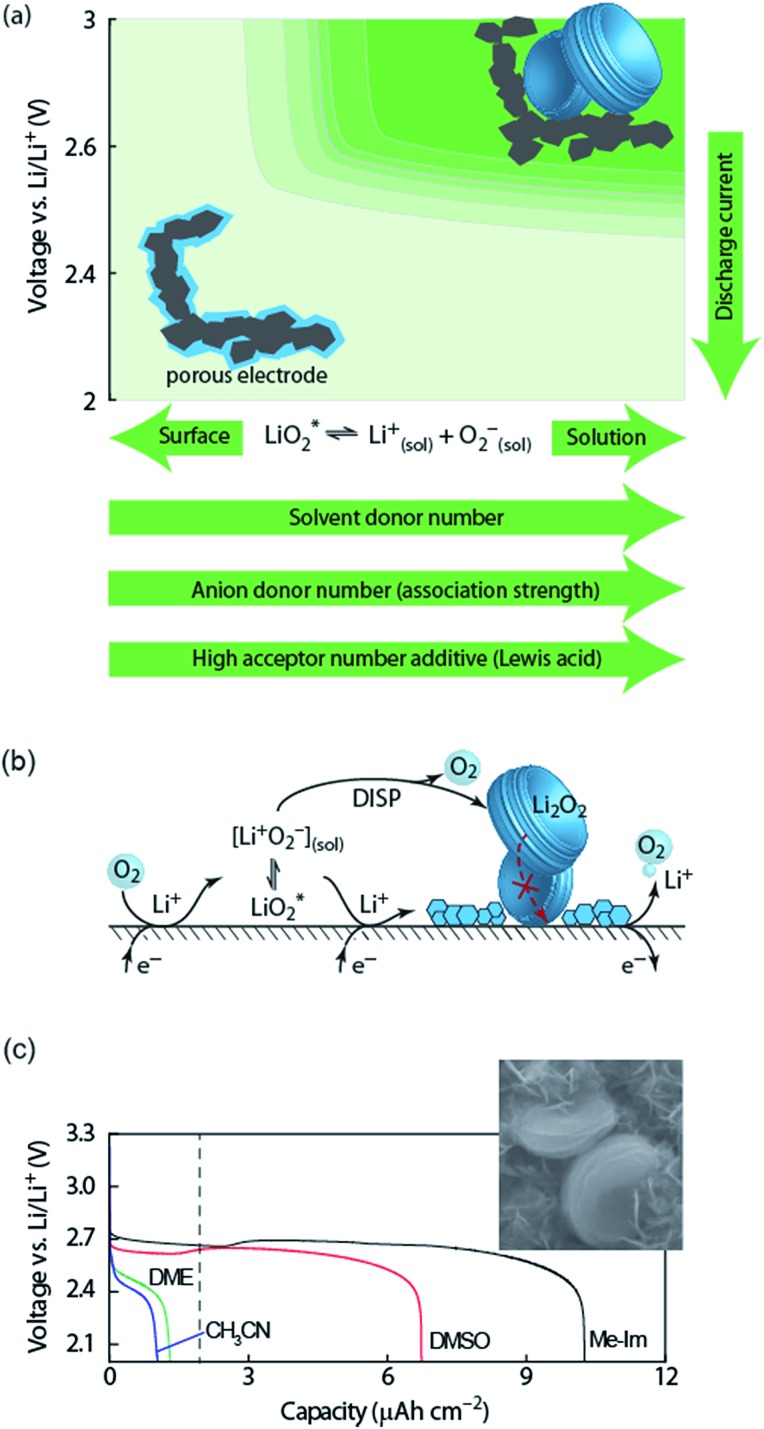
(a) Parameters determining surface and solution growth. These lead to Li_2_O_2_ as either conformal coating of the porous electrode or large particles in the pores. Effective Lewis basicity and acidity of the electrolyte as determined by solvent, salt anion, and additives governs the position of the equilibrium 
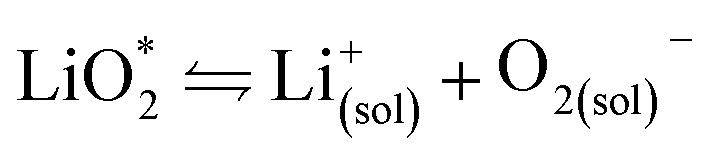
. Solvent and anion donor numbers follow the trend, *e.g.*, nitriles < glymes < amides < sulfoxide and TFSI^–^ < FSI^–^ < Tf^–^ < NO_3_^–^. High AN additives are, for example, H_2_O and alcohols. Increasing current shifts from solution to surface growth. (b) Reactions involved in the reduction mechanism and effect on charge. (c) Potential *versus* capacity for galvanostatic discharge in various electrolytes containing 0.1 M LiClO_4_. Me–Im is 1-methylimidazole (DN = 47). The dashed line indicates 7 nm solid layer thickness, which is seen as the limit for e^–^ tunneling. The insert shows an electron micrograph of toroidal deposits composed of lamellae as obtained from solution growth. (c) is adapted from ref. 11, the insert in (c) is reproduced from ref. 12 with permission from NPG.

The first step of O_2_ reduction in aprotic Li^+^-electrolytes results in superoxide (O_2_^–^), which associates with Li^+^ and in the second step either undergoes a second 1e^–^ reduction or disproportionates to form Li_2_O_2_. Two mechanisms have been proposed for how these steps proceed. The first involves a solution process, where O_2_^–^ is solubilized to precipitate Li_2_O_2_ from the electrolyte solution,^8^ and the second considers the intermediate as surface bound throughout the process.^9,10^ Recently, a unified mechanism was described, where the solution and surface mechanism, respectively, are limiting cases.^11^ It describes the partition between these cases by the solubilisation of LiO_2_ in the equilibrium1
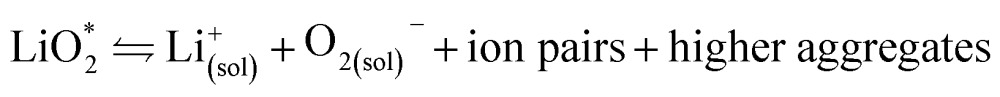
where * denotes surface species. In aprotic solvents the solubility of salts is primarily determined by the solvation of the cation, which is correlated with the Gutmann donor number (DN).^8,11,13,14^ O_2_^–^ solvation, correlated with the solvent acceptor number (AN), is usually weaker.^13^ The typical classes of electrolyte solvents span a wide range of DN from nitriles and sulfones (DN = 14–16), *via* glymes (DN = 20–24), amides (DN ∼ 26), sulfoxide (DN ∼ 30).^11,14^ Fig. 2a summarizes these and further parameters influencing surface or solution growth.

Lewis basicity (DN) or acidity (AN) of the electrolyte solution can be influenced by additives. Strongly Li^+^-coordinating salt anions can shift above equilibrium to the right to a similar extent as the solvent DN by competing association with the Li^+^ ion between salt anion and O_2_^–^.^15–18^ Similarly, Lewis acidic additives enhance O_2_^–^ solvation as shown with water, alcohols, and onium cations.^12,19–22^ Unfortunately, both high DN solvents and protic additives that favour the solution mechanism can enhance parasitic reactions.^12,20,23,24^

Irrespective of whether the surface or solution pathway prevails, the second electron transfer may either proceed *via* a second 1e^–^ reduction or disproportionation (Fig. 2b). With a standard potential of 2.96 V for O_2_/Li_2_O_2_ and ∼2.65 V for O_2_/O_2_^–^, the standard potential for O_2_^–^/Li_2_O_2_ is at ∼3.3 V.^11^ The second reduction has therefore at all discharge potentials a strong driving force. However, electrochemical measurements combined with *in situ* Raman have shown that disproportionation dominates at low overpotentials in electrolytes with sufficient solvation strength to support solution growth.^11,25,26^ Higher overpotential (higher current) accelerate in the same electrolytes the second reduction at the expense of disproportionation and change the mechanism to surface growth. Recently, it was suggested that not only effective solvation strength controls surface/solution growth based on a correlation between effective solvent polarity (*E*Nτ), tuned by additives, and the onset of the second reduction in DMSO.^27^ This was explained by an increasing solvent rearrangement energy with polarity and thus, according to Marcus–Hush theory, an increasing activation barrier for the second reduction.

Which mechanism prevails has important consequences for attainable capacity as exemplified in Fig. 2b for variation of solvent DN. The surface mechanism provides little mobility for reduced O_2_ species and leads to a conformal coating of the electrode with discharge ceasing after only ∼5 to 10 nm, corresponding to low capacity.^10,25^ Beyond this thickness the charge transport resistance increases greatly as determined by impedance spectroscopy and does not permit sustaining the current any longer.^10,28^ The solution mechanism, in contrast, keeps electrode area open for longer and allows for larger capacity by the growth of large (micrometer sized), toroidal particles composed of lamellae, which can fill larger pores to a bigger extent (insert in Fig. 2c).^25^ The electrochemically active surface area does initially not change, followed by gradual surface blocking. The capacity is equally limited by greatly increasing charge transfer resistance *R*_CT_.^28^ Concurrently, the aspect ratio and average particle size of newly formed Li_2_O_2_ decreases with progressive discharge.^29^ Together, evolution of *R*_CT_ and particle shape suggest that with shrinking active surface, growing overpotential and local current, the mechanism gradually shifts towards the surface mechanism, which finally causes full passivation.

### 3.2. Oxidizing Li_2_O_2_ on charge

Galvanostatic charging of Li–O_2_ cathodes is typically characterized by an onset of charging (O_2_ evolution) slightly above the OCV at ∼3 V and ever increasing voltage as charging progresses. Three underlying phenomena appear to be consolidated although details are still under debate: (1) electrochemical oxidation of Li_2_O_2_ is possible with low kinetic barriers at high rates; (2) increasingly difficult electron transfer along recharge contributes a minor fraction of the voltage rise; (3) rising voltage is mostly caused by accumulating parasitic products, which cause a mixed potential.

**Fig. 3:**
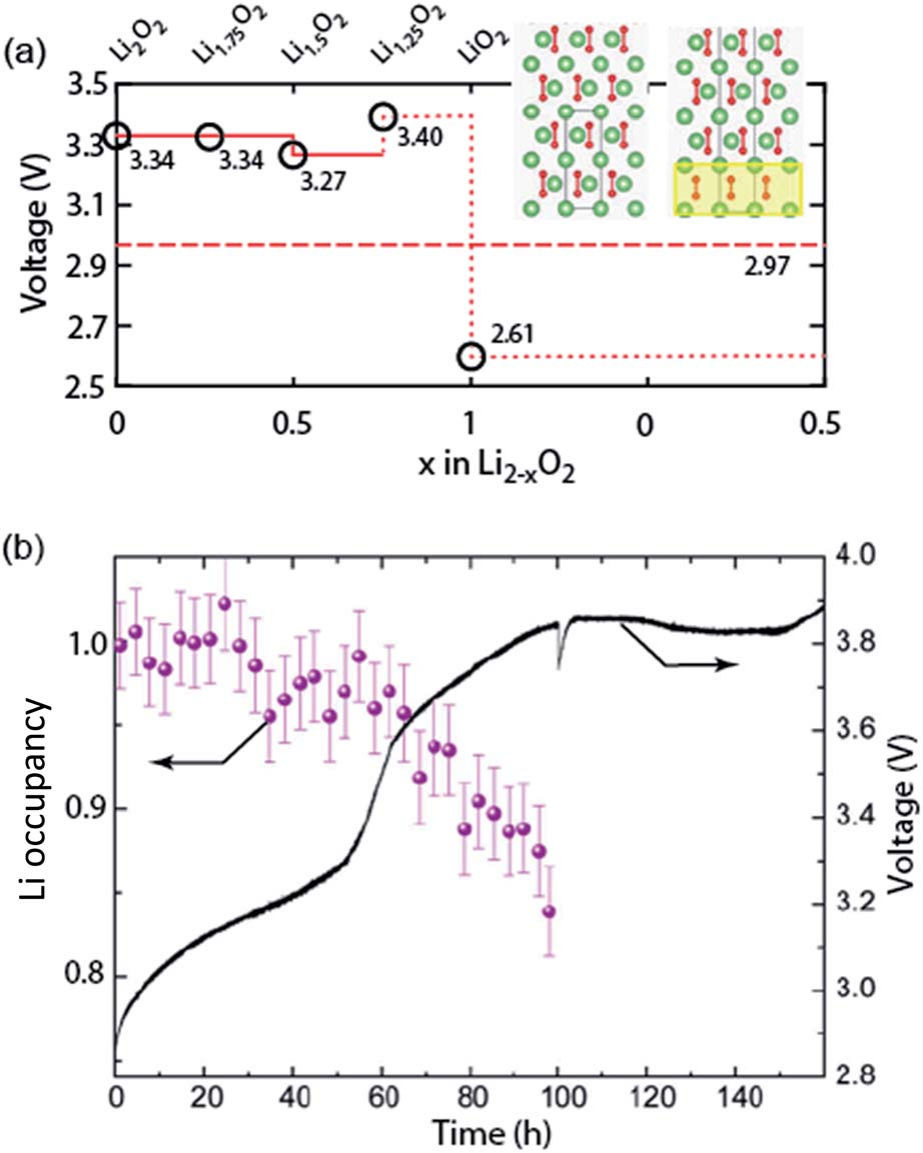
(a) Calculated oxidation potentials for topotactic delithiation of Li_2_O_2_ to Li_2–*x*_O_2_. The dashed line denotes the O_2_/Li_2_O_2_ standard potential. The inserts show the structures of Li_2_O_2_ and Li_1.75_O_2_.^32^ (b) Average Li occupancy during charging of electrochemically formed Li_2_O_2_ and the associated voltage.^35^ (a) and (b) are adapted from ref. 32 and 35, respectively, with permission from the American Chemical Society.

Theoretical studies determined the overpotential at which Li^+^, e^–^, and O_2_ can be removed from Li_2_O_2_.^30–33^ They suggested that Li^+^ and e^–^ can be removed starting below 0.2 V overpotential, leading to either surface 
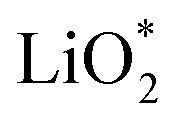
 or bulk Li_2–*x*_O_2_ *via* topotactic delithiation.^30–32^ Bulk Li_2–*x*_O_2_ appears to comprise Li_2_O_2_ and LiO_2_ domains.^32^ O_2_ evolution was initially suggested to have the highest barrier along the whole path.^30,31^ Later, a pathway was shown where O_2_ evolves facile after progressive delithiation at ∼0.3 V overpotential *via* Li_2–*x*_O_2_ to LiO_2_, which then decomposes rapidly at ∼2.6 V *vs.* Li/Li^+^ (Fig. 3a).^32^ Low theoretical charge overpotential is in agreement with experiments, albeit experimental overpotential even approaches zero since observed O_2_ evolution starts from ∼3 V.^9,33,34^ Li-deficient Li_2–*x*_O_2_ phases were confirmed by operando X-ray diffraction (Fig. 3b).^35^ An open question is whether O_2_ release after the initial delithiation proceeds by disproportionation of LiO_2_ domains in Li_2–*x*_O_2_ *via* 2LiO_2_ → Li_2_O_2_ + Li^+^ + O_2_ or *via* further e^–^ extraction. This is significant as the first pathway would imply that charge could be influenced in much the same way as discharge by the discussed factors governing surface or solution routes, and it could be a key for singlet oxygen formation and thus the major source a parasitic chemistry on charge as discussed in Section 4.3.

An intriguing feature of Li_2_O_2_ is that the electronic conductivity depends strongly on applied voltage, crystallinity and defects. Increasing potential was postulated to significantly increase conductivity by either reducing the tunnelling barrier or through the formed Li-deficient phases.^36,37^ This is consistent with impedance measurements that reveal much higher, capacity limiting, polarization resistance at the end of discharge than after switching to charging.^28^ Theoretical and experimental work on pure Li_2_O_2_ have highlighted the importance of defects and grain boundaries on charge transport.^38–40^ Orders of magnitude higher conductivity of amorphous *vs.* crystalline Li_2_O_2_ is consistent with the observed oxidation of amorphous and poorly crystalline Li_2_O_2_ at lower voltage followed by the crystalline Li_2_O_2_.^35,38,41^

Important for understanding polarization and limitations of recharge is whether oxidation occurs at the cathode/Li_2_O_2_ or the Li_2_O_2_/electrolyte interface. While charge transport through Li_2_O_2_ will be limiting in the latter case, in the former case the question arises whether Li_2_O_2_ particles would lose contact, which would impede full recharge. Recent work including with isotope labelled O_2_ suggested that e^–^ transport is limiting during discharge and charge.^9,42^ Thus, Li_2_O_2_ can deposit at the cathode/Li_2_O_2_ interface beneath previously formed product if Li^+^ and O_2_ can reach the surface *via* cracks in the Li_2_O_2_ and this later deposit is oxidized first, eventually leading to a gap between cathode and Li_2_O_2_ and contributing to increasingly difficult oxidation.^42^ Note that the associated rise in impedance accounts only partly for the observed rise in charge potential. The majority of the rise is associated with concomitant parasitic chemistry from the start of charge, which is accelerated with growing potential and predominantly caused by singlet oxygen as discussed in Section 4.3.^24,43–45^ Current understanding of solid catalysts for the OER will be discussed in Section 5.2 and redox mediators as charge transfer agents for O_2_ reduction and evolution in Section 6.

## 4. Parasitic chemistry

Typically close to two electrons per one O_2_ are consumed on discharge despite significant amounts of a typical pattern of side products being formed including Li_2_CO_3_, Li formate and Li acetate.^46–49^ On charge, the e^–^/O_2_ ratio typically deviates significantly from two and more of the side products form. Parasitic chemistry is the prime obstacle for reversible Li–O_2_ cell cycling and understanding the mechanisms to counteract them is thus the most pressing research need in the field.

### 4.1. Characteristics of reversible cell reactions

True reversibility of the cathode reaction, 2Li^+^ + O_2_ + 2e^–^ ↔ Li_2_O_2_, requires a set of quantities to obey the stoichiometry and to match each other during discharge and subsequent charge. These are:

(A) One mole of O_2_ is consumed/released per two moles of electrons flowing on discharge/charge. Thus e^–^/O_2_ = 2.

(B) One mole of O_2_ and two moles e^–^ produce exactly one mole of Li_2_O_2_ on discharge. On charge two moles e^–^ consume one mole of Li_2_O_2_ and release one mole of O_2_. Thus:

2 e^–^/O_2_ = e^–^/Li_2_O_2_ = 2 and O_2_/Li_2_O_2_ = 1

The ratio e^–^/O_2_ = 2 is not a strict requirement for a rechargeable Li–O_2_ battery if Li_2_O_2_ is not the discharge product as occasionally claimed.^6,7,50^ For example, if Li_2_O_2_ with a certain fraction of ‘LiO_2_-like’ species or even pure LiO_2_ is the product e^–^/O_2_ may be lower than 2. However, in any case e^–^/O_2_ = e^–^/Li_*x*_O_2_ must be identical on discharge and charge. These conditions further imply:

(C) All electrons involved contribute to the oxygen reduction reaction (ORR) or oxygen evolution reaction (OER). Thus, no other gas than O_2_ evolves during discharge and charge and no soluble or solid product other than Li_2_O_2_ (or H_2_O_2_) is produced.

(D) For cycling with equal capacity on discharge and charge (*Q*_ORR_ = *Q*_OER_) the O_2_ released on charge matches the amount consumed, thus *n*_O_2_,ORR_ = *n*_O_2_,OER_.

Importantly, none of these measures can be taken for granted to be mutually met even if, for example, e^–^/O_2_ ≈ 2 on discharge is fulfilled. Before discussing the current understanding of reactions leading to deviating measures in the next two subsections, we first consider the basic interpretation of the load curves and quantitative analyses to determine the measures (A) to (D).

**Fig. 4:**
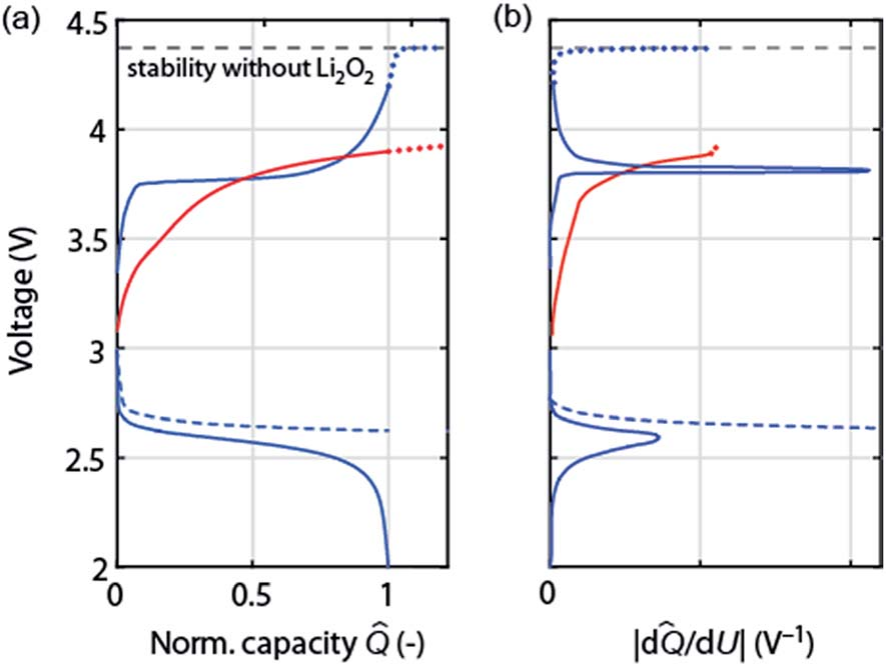
(a) Schematic of load curves that are either possibly commensurate with cycling according to Li_2_O_2_ → O_2_ + 2Li^+^ + 2e^–^ (blue) or with certainty indicating a major fraction of parasitic chemistry (red). The left graph shows the voltage *versus* normalized capacity *Q* (capacity divided by final discharge capacity). Full and dashed curves correspond to voltage or capacity controlled discharge, respectively. Dotted curves on charge extrapolate to overcharge. (b) The corresponding differential capacity curves |d*Q*/d*U*|.

As discussed in Section 2 on performance metrics, it has become a habit to cycle cells at fixed discharge/charge capacities of, *e.g.*, 1000 mA h g_substrate_^–1^, thus forcing *Q*_charge_ = *Q*_discharge_. Truncating discharge reasonably to avoid full electrode blockage may enable cyclability and appears justified as long as the true capacity based on the total weight is obeyed (see Fig. 1a). Capacity controlled recharge with *Q*_charge_ = *Q*_discharge_ is, however, prone to mask parasitic chemistry as illustrated in Fig. 4. The full and dashed blue curves at the bottom show discharge with voltage or capacity limitation, respectively. Basic thermodynamics requires for the charge reaction to be the reverse of the preceding discharge that (a) the voltage remains within the stability of electrolyte and electrode without Li_2_O_2_ (black dashed line); and (b) that as the capacity approaches full recharge the depletion of the Li_2_O_2_ must cause the voltage to rise ever steeper before it transits into a plateau at the electrolyte oxidation potential, blue curve in Fig. 4a. Concurrently, differential capacity d*Q*/d*U* must approach zero at full recharge irrespective of whether the preceding discharge was limited by voltage or capacity, Fig. 4b (blue curve).

Frequently observed load curves of the type as shown in the red curve in Fig. 4 are, in contrast, with certainty indicating major parasitic chemistry. They are characterized of approaching full recharge flat with d*Q*/d*U* remaining high, Fig. 4a and b. Flat and high relate here to the rest of the charge curve. Whatever reaction takes place can thus not have finished, can thus not be predominantly Li_2_O_2_ oxidation, and would continue with a continuing flat plateau if such a cell were over charged (extrapolation by the red dotted curve). An exception is cells with oxidation mediators with large electrolyte-to-Li_2_O_2_ ratio. Notably, remaining within the oxidative stability window (without Li_2_O_2_) does not ensure absence of oxidative electrolyte decomposition nor does a lower recharge plateau *per se* (*e.g.*, as a result of electrocatalysts) indicate less parasitic chemistry.^47,51^

Concluding about reversibility by the measures (A) to (D) requires multiple quantitative analyses. Measuring O_2_ consumption/evolution has been described by two methods: (A) quantitative operando online mass spectrometry (MS), where the cell head space is continuously or intermittently purged to a MS.^34,48,52^ Using an O_2_/Ar mix allows also quantification of O_2_ consumption and of any other gases evolved on discharge.^19,24,34,53^ (B) Measuring the pressure in a closed cell head space.^54^ Peroxide or superoxide content of electrodes has been measured *ex situ* using either iodometric titration^49^ or spectrophotometry using the coloured [Ti(O_2_^2–^)]^2+^ complex.^55^ This method was combined with equally MS based quantification of Li_2_CO_3_ and organic products by treatment with acid and Fenton's reagent to separately evolve CO_2_ from inorganic and organic compounds.^44^ The latter may also be quantified by ^1^H-NMR after immersing the electrode in D_2_O which further allows for speciation of the compounds.^47,49^ Importantly, all these methods capture the integral electrode. Qualitative spectroscopic or microscopic methods such as Raman, FTIR, XRD, XPS, or SEM cannot replace the mentioned or similar quantitative integral methods and cannot support claims of reversibility.

### 4.2. Reactions with reduced oxygen species and molecular oxygen

Reduced reactive oxygen species (RROS) are well known for their reactivity with a wide range of organic substrates, which has both been used as a reactant and recognized as a source of unwanted reactions.^56,57^ Primary species are O_2_^–^ and O_2_^2–^, which in protic environments form species including HOO˙, HOO^–^, and HO˙. Their nucleophilicity, basicity, and/or radical nature cause reactivity *via* three major routes: nucleophilic substitutions, H^+^ and H-atom abstraction. The latter may also proceed with dioxygen in auto-oxidation reactions. Additionally, O_2_^–^ can transfer electrons.

Polarity is introduced to aprotic electrolyte solvents *via* heteroatoms to dissolve a Li-salt. The polarity in turn makes adjacent C and H atoms reactive. After the complete failure of carbonate electrolytes for Li–O_2_ chemistry was rightly associated with the nucleophilicity of O_2_^–^, this and the other reactivities of RROS and O_2_ were taken to explain lesser but significant parasitic chemistry of all so far investigated alternative solvents and cell components.^1,47,58^ In the era of computational chemistry the likelihood for this assignment can be judged based on activation and reaction free energies. Bryantsev *et al.* have pioneered this field for O_2_^–^ and O_2_ reacting with organic electrolytes *via* nucleophilic substitution, H-atom, and H^+^ abstraction.^58–61^ Their data for activation energies together with those of other researchers are summarized in Table 1.

**Table 1:** Reactions of organic electrolytes with reduced oxygen species and molecular oxygen and their calculated activation energy barriers. ROR′ is generically used for organic moieties with polarizing heteroatoms and reactions may accordingly be translated to, *e.g.*, N or S containing ones

Reactant	Type of reaction	Reaction	*E* ^act^ (kJ mol^–1^)	References
O_2_^–^	Nucleophilic substitution	ROR′ + O_2_^–^ → RO^–^ + ROO˙ (3)	121–144^*a*^, 105^*b*^, 65–95^*c*^	58, 61, 65 and 66
	H-atom abstraction	RH + O_2_^–^ → R˙ + HOO^–^ (4)	129–180^*d*^, 191^*e*^	61, 63, 65 and 67
	H^+^ abstraction	RH + O_2_^–^ → R^–^ + HOO˙ (5)	p*K*_a_ > 30 stable^*f*^	59 and 68
Li_2_O_2_	Nucleophilic substitution	ROR′ + Li_2_O_2_ → RO^–^Li^+^ + R′OO^–^Li^+^ (6)	134–192^*a*^	62
	H-atom abstraction	RH + Li_2_O_2_ → R˙ + [Li_2_O_2_-H˙] (7)	96–112^*a*^	62
	H^+^ abstraction	RH + Li_2_O_2_ → R^–^Li^+^ + HOO^–^Li^+^ (8)	116–311^*a*^	62 and 66
O_2_	H-atom abstraction	RH + O_2_ → R˙ + HOO˙ (9)	163–183^*g*^, 138–161^*h*^	61

^*a*^Dimethoxyethane (DME).

^*b*^Acetonitrile.

^*c*^Carbonate and lactones.

^*d*^Free DME.

^*e*^The DME_2_–Li^+^ complex.

^*f*^Examples for p*K*_a_ < 30: –CH_2_–CF_2_–, polyvinylidene difluoride (PVDF), aliphatic dinitriles, alkyl imides. p*K*_a_ > 30: acetonitrile, DMSO, *N*-alkyl amides and lactams, aliphatic ethers.

^*g*^The lower value for free DME, the higher one for the DME_2_–Li^+^ complex.

^*h*^Lactams and amides.

Strikingly, activation energies for all considered reactions involving the major classes of solvents – except for the very unstable solvents like carbonates – are too high to expect these reactions to strongly contribute to decomposition. Based on solvent stability screening experiments with KO_2_ exposure or the reversibility of the O_2_/O_2_^–^ couple, reactions with activation energies beyond 100 kJ mol^–1^ can be considered not to contribute noticeably.^58^ Hence, only esters and lactones are expected to react *via* nucleophilic substitution with O_2_^–^ and possibly ethers *via* H-abstraction with Li_2_O_2_.^62^ With ethers, for example, all pathways with O_2_^–^, Li_2_O_2_ and O_2_ require high activation energy and are strongly endothermic. The only exception is one study that found H-abstraction by Li_2_O_2_ clusters slightly exothermic with *E* ^act^ < 100 kJ mol^–1^.^62^ Solvent coordination with Li^+^ was reported to further stabilize against H-abstraction by O_2_^–^ and O_2_.^61,63^

In presence of proton sources such as water or weak acids O_2_^–^ forms *via*eqn (10)–(13) HOO˙, HOO^–^, and HO˙, which are more reactive than the primary RROS.^59,64^

10 O_2_^–^ + H^+^ → HOO˙

11 HOO˙ + O_2_^–^ → HOO^–^ + O_2_

12 2HOO˙ → H_2_O_2_ + O_2_

13 HOO^–^ + H_2_O_2_ → O_2_^–^ + HO˙ + H_2_O

HOO^–^ is a stronger base than O_2_^–^ and more readily abstracts protons to form R^–^. HO˙ could serve as the initiator to form R˙, which undergoes fast and thermodynamically favourable onwards chain reactions in the presence of O_2_.^47,61^ Overall, direct reactivity of O_2_^–^, Li_2_O_2_ and O_2_ with the most important classes of non-aqueous solvents for the Li–O_2_ cathode is unfavourable. Increasing parasitic chemistry with increasing water content is consistent with the protonated species being more reactive.^12^ Yet, much higher side reactions on charge than on discharge, which opposes superoxide occurrence, points at RROS not to be the prime cause for parasitic chemistry.

### 4.3. Singlet oxygen formation and suppression during discharge and charge

Electrochemically oxidizing Li_2_O_2_ was hypothesised by Hassoun *et al.* to be able to generate singlet oxygen (^1^Δ_g_ or ^1^O_2_), the highly reactive first excited state of triplet ground state dioxygen (^3^Σ_g_^–^ or ^3^O_2_).^69^ This view was motivated by the known formation of ^1^O_2_ by chemical oxidation of H_2_O_2_ or alkaline peroxides.^70^ Based on the reversible potential of Li_2_O_2_ formation and the energy difference between triplet and singlet oxygen of ∼1 eV, ^1^O_2_ formation in the Li–O_2_ cell has been considered possible at charging potentials exceeding 3.5 to 3.9 V *vs.* Li/Li^+^.^54,69,71^ The idea was picked up by several reports but could not be verified due to the difficulties with detecting ^1^O_2_ except for one work, which identified small quantities of ^1^O_2_ between 3.55 and 3.75 V and explained it on thermodynamic grounds for the process Li_2_O_2_ → O_2_ + 2Li^+^ + 2e^–^.^18,45,71^ It could thus contribute to explaining parasitic chemistry above 3.55 V. Yet it has been found that from the start of charging below 3.5 V both a substantial amount of parasitic products is generated^43,44,49^ and that less than 1 mol O_2_ evolves per 1 mol Li_2_O_2_ consumed.^49^ Both could not be explained by reactivity of reduced O_2_ species and formation of ^1^O_2_ above 3.55 V.

**Fig. 5:**
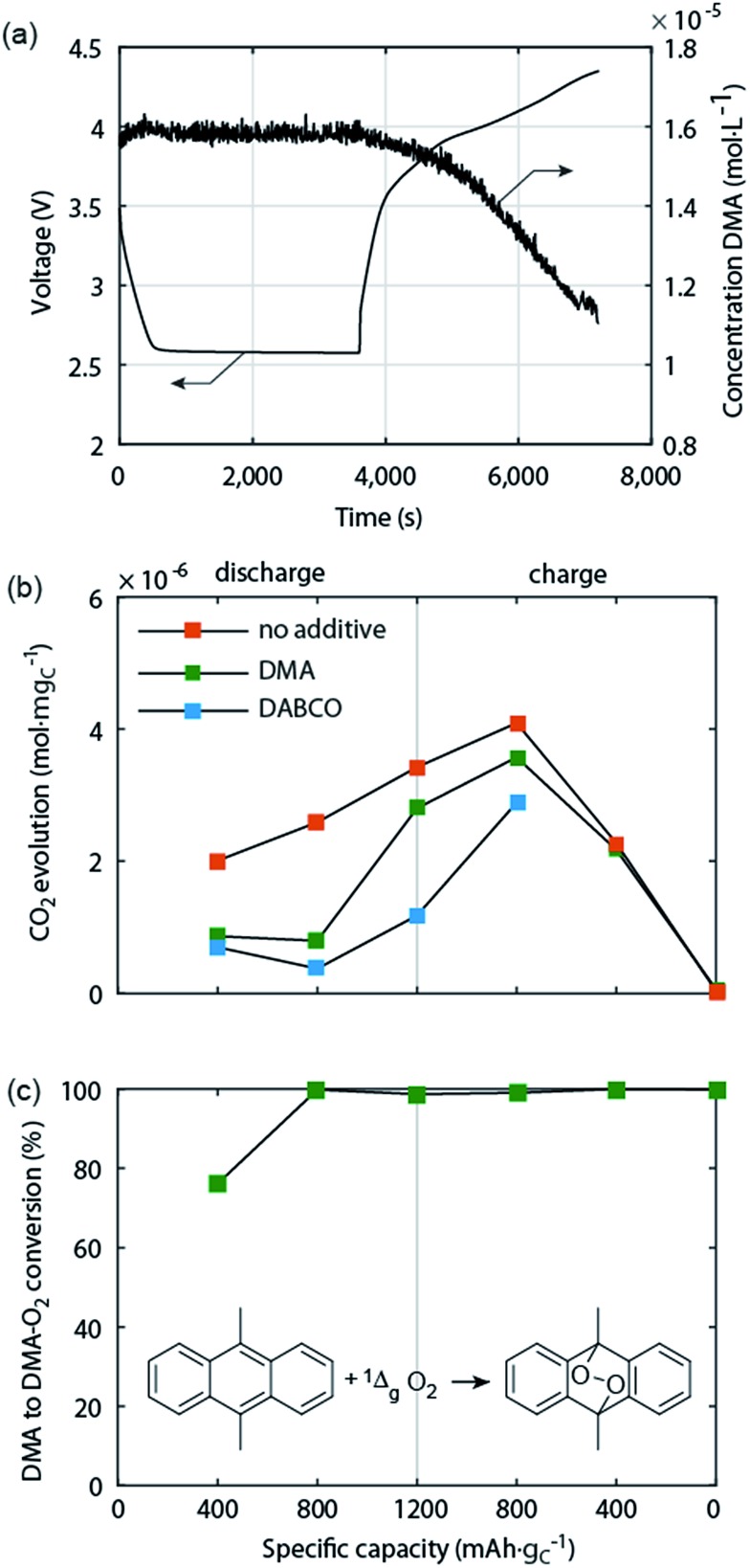
Singlet oxygen formation and suppression during cycling of the Li–O_2_ cathode.^24^ (a) Operando fluorescence spectroscopy during galvanostatic discharge and charge of a carbon black electrode in O_2_ saturated 0.1 M LiClO_4_ in tetraglyme containing 1.6 × 10^–5^ M 9,10-dimethylanthracene (DMA) as singlet oxygen trap. (b) Amount of carbonaceous side reaction products at various sample points during discharge and charge of cells containing no additive, 30 mM trap DMA, or 10 mM quencher DABCO. (c) Fraction of the initial DMA that has reacted to DMA–O_2_ in the cells that contained DMA as additive.

Recently, Mahne *et al.* have shown that ^1^O_2_ forms also during discharge and from the onset of charge and that it accounts for the majority of parasitic reaction products.^24^ The amount of ^1^O_2_ increases during discharge, early stages of charge, and charging at higher voltages, and is enhanced by the presence of trace water. They used the ^1^O_2_ specific conversion of 9,10-dimethylanthracene (DMA) into its endoperoxide (DMA–O_2_) to probe ^1^O_2_ in the cell. Operando fluorescence detection on discharge and charge has shown rather small ^1^O_2_ abundance on discharge and significant ^1^O_2_ formation immediately after switching to charging, starting from ∼3 V (Fig. 5a). ^1^O_2_ on discharge is significant as shown by detecting the degree of DMA to DMA–O_2_ conversion (Fig. 5c) and the substantially reduced amount of side products with DMA (Fig. 5b). Hence, a suitable ^1^O_2_ trap such as DMA can divert ^1^O_2_ from reacting with cell components, is, however, quickly consumed at the level of ^1^O_2_ abundance. In contrast, a ^1^O_2_ quencher physically deactivates singlet into triplet oxygen and is itself not consumed. Using 1,4-diazabicyclo[2.2.2]octane (DABCO) as quencher they have shown even more substantial reduction of side products (Fig. 5b). DABCO has, however, limited electrochemical stability between ∼2.0 and 3.6 V, which allows for only partial recharge. Future work should therefore focus on finding quenchers that meet all requirements including electrochemical potential window, stability with the reduced oxygen species, and high quenching rate.

On discharge one possible source of ^1^O_2_ is the disproportionation of LiO_2_ according to

14 2LiO_2_ → (LiO_2_)_2_ → Li_2_O_2_ + ^1^O_2_

This pathway appears plausible when the structures and energies of dimers as calculated by Bryantsev *et al.* are considered.^60^ When H_2_O or other proton sources are available the superoxide will be protonated to form HOO˙ that has been reported to be able to release ^1^O_2_ in the overall reaction.^72,73^

15 2O_2_^–^ + 2H^+^ → H_2_O_2_ + ^1^O_2_

Overall, the disproportionation of superoxide in the presence of either Li^+^ or H^+^ appears to be the ^1^O_2_ source on discharge. On charge three possible pathways were suggested. First, an analogous path to the one on discharge involving disproportionation of superoxide in the presence of either Li^+^ or H^+^. The LiO_2_-like surface species could form during the initial charging steps as discussed above^30–32^ and ^1^O_2_ may form analogously to eqn (14) or (15). This pathway for ^1^O_2_ formation can be active from the first onset of charge as soon as Li^+^ and e^–^ are extracted. Second, a further 1e^–^ oxidation of the surface LiO_2_ species could give ^1^O_2_ above 
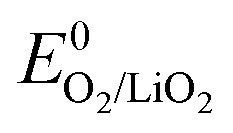
 + *E*(^1^Δ_g_ ← ^3^Σ_g_^–^). With the thermodynamic equilibrium potential 
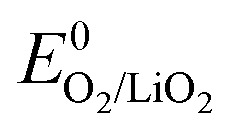
 estimated to be between 2.29 and 2.46 V (ref. 32, 74 and 75) and *E*(^1^Δ_g_ ← ^3^Σ_g_^–^) ∼ 1 eV, a thermodynamic voltage for ^1^O_2_ evolution of 3.26 to 3.43 V can be estimated. Finally, above ∼3.55 V the pathway can sets in as suggested by Hassoun *et al.* and shown by Wandt *et al.* with ^1^O_2_ evolving by 2e^–^–oxidation of Li_2_O_2_ (Li_2_O_2_ → O_2_ + 2Li^+^ + 2e^–^).^69,71^

Superoxide is both a proficient source and efficient quencher of ^1^O_2_ *via* eqn (5).^76^

16 O_2_^–^ + ^1^O_2_ → ^3^O_2_ + O_2_^–^

Net formation of ^1^O_2_ may depend on the relative kinetics of all superoxide sources and sinks (with ^1^O_2_ being involved in both) and not solely on the superoxide concentration. These sources and sinks are both electrochemical and chemical and change with discharge/charge, electrolyte, current, and potential. Current density and electrolyte properties will influence the ^1^O_2_ formation in much the same way as they govern the occurrence of superoxide on discharge and charge below 3.5 V.^77^ Charge current will drive ^1^O_2_ production if it causes charging voltages above ∼3.5 V.

### 4.4. Alternative storage media to Li_2_O_2_

In occasional reports the discharge product was reported to be Li_2_O_2_ with remaining stable LiO_2_ species, resembling the Li_2–*x*_O_2_ intermediate on charge.^50^ Based on these findings an Ir–graphene based cathode was reported to cycle in ether electrolyte *via* crystalline LiO_2_.^7^ These assignments were mostly based on Raman spectra that can distinguish O–O stretch vibrations in Li_2_O_2_ and LiO_2_. However, it was recently shown that PVDF binder decomposition can lead to vibrations mimicking those of LiO_2_, thus concluding that cycling was not based on LiO_2_.^78^

Surprisingly large water contamination up to 1000s of ppm in ether electrolyte has been shown to still lead to Li_2_O_2_ as the main discharge product rather than LiOH as one could intuitively assume.^12,19^ Instead, water promotes discharge *via* the solution mechanism. Whether Li_2_O_2_ or LiOH forms was suggested to be governed by the effective p*K*_a_ value of water in the electrolyte.^20^ A value of 35 in MeCN compared to 47 in DME was used to explain LiOH to form in the former and Li_2_O_2_ in latter. At water concentrations beyond 1% LiOOH was shown to form together with LiOH.^79^ LiOH was also found to form in a 4e^–^/O_2_ reduction in presence of LiI.^6,80^ Unfortunately, O_2_ evolution from LiOH could so far not be shown and apparent cyclability must be accounted for I^–^ electrochemistry and parasitic chemistry.^80^

Li_2_CO_3_ not only forms as a result of parasitic reactions, but also when the O_2_ supply is CO_2_ contaminated.^47,81,82^ CO_2_ has a high barrier for direct reduction but reacts readily with O_2_^–^ along with the formation of Li_2_O_2_. If Li_2_CO_3_ could be decomposed on charge to the educts it would make the cell insensitive to CO_2_ in the O_2_ supply. While Li_2_CO_3_ can be decomposed from ∼3.8 V, it does not evolve O_2_ together with the CO_2_, which suggests that reactive intermediate form that decompose the electrolyte.^47,48,82^ Apparent cyclability of O_2_/CO_2_ cells that was shown in some cases was so far not compellingly associated with reversible chemistry. Making Li–O_2_ chemistry insensitive to H_2_O and CO_2_ contamination should thus remain a high priority, which foremost calls for rigorously investigating the associated parasitic chemistry.

## 5. Porous cathode design

### 5.1. Cathode support

Carbon is the most common porous electron conducting matrix for the O_2_–cathode due to low cost, high conductivity, and easily tuneable surface area and pore sizes. Carbon was, however, found to decompose itself on cycling and to promote electrolyte decomposition.^43,44,49,83^ Using ^13^C carbon black and MS analysis of the gaseous and solid products at various stages of cycling, carbon was observed to be relatively stable on discharge despite thermodynamic instability in contact with Li_2_O_2_; the majority of side products stems from the electrolyte. From the onset of charge, however, carbon decomposes to form Li_2_CO_3_ with increasing rate as the potential grows.^44,49^ Defect rich hydrophilic carbon is both much more vulnerable itself and promotes more strongly electrolyte decomposition during discharge and charge than hydrophobic carbon.^44,83^

As with electrolyte decomposition, the carbon instability was related to O_2_^–^ attack. The perfectly opposing trend of O_2_^–^ abundance – highest on discharge and ever decreasing as charge voltage grows – to decomposition rates makes this interpretation unsatisfactory. Therefore, reactive intermediates on oxidizing Li_2_O_2_ have been suggested.^44,45^ Carbon and electrolyte decomposition rates both follow the trend of ^1^O_2_ abundance as shown in Fig. 5a. This is consistent with ^1^O_2_ being the dominant driver of parasitic chemistry; possibly the nearly exclusive one on charge.

Given the instability of carbon, alternative corrosion resistant materials have been sought that at the same time do not promote electrolyte decomposition. They include Ti ceramics and nanoporous Au that allow for more stable cycling.^52,53^ For TiC a thin passivating layer of TiO_2–*x*_ and TiOC has been identified to be critical for stability and conductivity.^53,84^ The metallic Magnéli phase Ti_4_O_7_ was equally shown to form surface TiO_2–*x*_ and to allow for cyclability similar to Au and TiC.^85^ Limited binder stability adds another dimension to cathode design. Standard PVDF binder as used in LIB was found to react with Li_2_O_2_ and KO_2_,^59,78,86,87^ but equally ^1^O_2_ can be expected to contribute. More stable alternatives include PTFE and Nafion.^86,87^

**Fig. 6:**
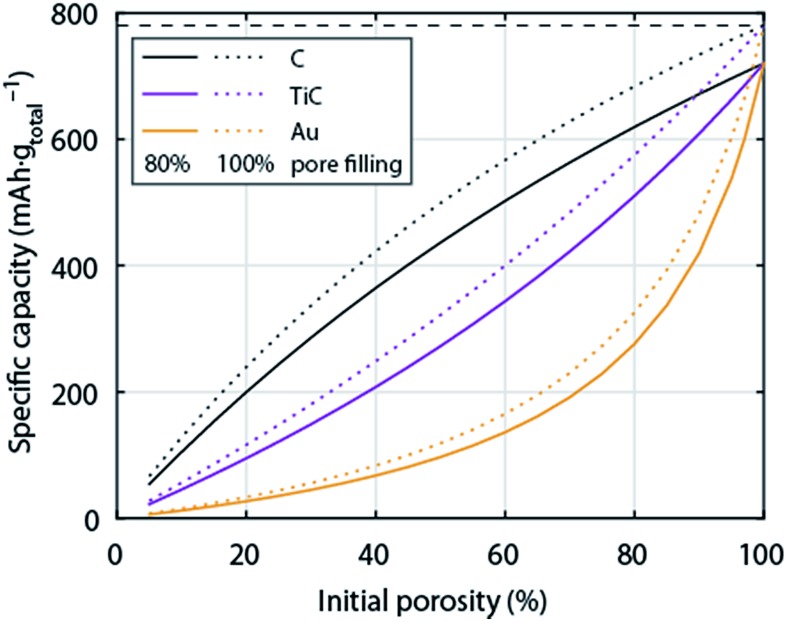
Specific capacity of a Li–O_2_ cathode with respect to total electrode weight including electrolyte as a function initial porosity. The initial porosity is in the fully charged state filled with electrolyte and at full discharge filled to 80% with Li_2_O_2_; the dotted lines show values for 100% pore filling for comparison. Values are given for cathodes made from C, TiC, or Au respectively. The calculation is analogous to Fig. 1.

So far the surface chemistry and electrochemistry of these alternative materials have been investigated with bound nanoparticles forming low porosity electrodes. Achieving high capacity based on the total electrode weight requires, however, filling highly porous electrodes to a large extent with Li_2_O_2_. High porosity becomes even more important when going from C to much denser metals or ceramics. Fig. 6 shows the relation between initial electrode porosity and maximum achievable true capacity for the examples of C, TiC, and Au. Shaping chemically stable materials into highly porous electrodes, ideally with well beyond 80% porosity, favourable surface area and pore size distribution arises therefore as a major need in the field.

### 5.2. Heterogeneous electrocatalysis

Typical overpotentials relative to 
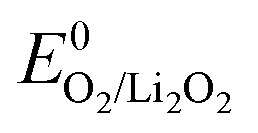
 = 2.96 V are ∼0.3 V on discharge and ever rising values on charge from nearly zero to, in some cases, up to 2 V. Inspired by aqueous O_2_ electrochemistry, these overpotentials were accounted to sluggish kinetics and evoked substantial efforts in finding efficient electrocatalysts including noble metals, transition metal oxides, and doped carbons.^51^ Considering the mechanism for Li_2_O_2_ formation/decomposition, there are, however, three major barriers questioning true effectiveness of solid electrocatalysts. First, ideal Li–O_2_ chemistry does not involve O–O bond breaking, the step necessitating catalysts in aqueous media. O_2_ reduction to superoxide and peroxide, in contrast, has been shown to be facile even on bare glassy carbon.^33^ Hence, perceived overpotentials are not due to ORR or OER kinetics. The discharge potential of ∼2.7 V is not due to overpotential of the O_2_/Li_2_O_2_ couple but is pinned there by the reversible potential of the O_2_/O_2_^–^ couple, the first step on O_2_ reduction to Li_2_O_2_ and can thus not be raised by electrocatalysts.^9,11,88^ Li_2_O_2_ oxidation starts with nearly zero overpotential at ∼3 V. Rising charge potentials are, as discussed in Section 3.2, caused partly by increasingly difficult electron transfer in the receding Li_2_O_2_ and mostly by parasitic chemistry. Second, conventional electrocatalysis would only act on dissolved redox species. On discharge there would thus be no effect beyond monolayers of Li_2_O_2_ forming with the surface mechanism. Deep discharge with both surface and solution mechanism blocks eventually all electrochemically active surface. Third, charge transport limitations through forming Li_2_O_2_ (ref. 10 and 36) and mass transport of O_2_ through the porous electrode^89^ are not addressed by solid catalysts.

Whether solid electrocatalysts can have any effect on charge depends on the processes at the buried cathode/Li_2_O_2_ interface and how they proceed as charge progresses. Given the insolubility of Li_2_O_2_, solution transport to open sites can be largely neglected. Proposed pathways for how the substrate could modulate oxidation of adhering Li_2_O_2_ include *in situ* doping of the deposited Li_2_O_2_ with slightly soluble transition metal catalysts during discharge, helping charging through enhanced polaron transport or vacancy transport with O_2_ evolution at the Li_2_O_2_/electrolyte interface.^90^ Further, supporting metal(oxides) may alter the delithiation kinetics of Li_2_O_2_ by forming Li-transition metal-oxides.^77^ Acting beyond the initial stages of charge requires maintaining the electrode/Li_2_O_2_ contact, for which the driving force is not clear as the Li_2_O_2_ closest to the electrode will necessarily be oxidized first. In the case of large Li_2_O_2_ deposits forming by the solution mechanism, this contact may never be fulfilled for a large fraction of the Li_2_O_2_. Not least did the habit of extended cycling at a small fraction of a possible single discharge capacity (see Section 2) arise from catalyst studies; with deep discharge the same cells fail typically very quickly, which hints at very limited effectiveness of the catalysts to oxidize large amounts of detached Li_2_O_2_.

Given the paramount importance of parasitic chemistry, electrocatalysts must not catalyse parasitic reactions with the electrolyte or electrode. Unfortunately, materials identified as electrocatalysts do enhance parasitic reactions.^46,51,91^ The exact pathways are not fully clarified but are at least in part associated with the catalyst's ability to dissociate the O–O bond. Concluding about a catalyst's ability to enhance efficiency and cyclability requires quantitative measures of reactant turnover and parasitic products without which any claim is inadequate (see Section 4.1).

## 6. Solution based Li–O_2_ cell chemistry

**Fig. 7:**
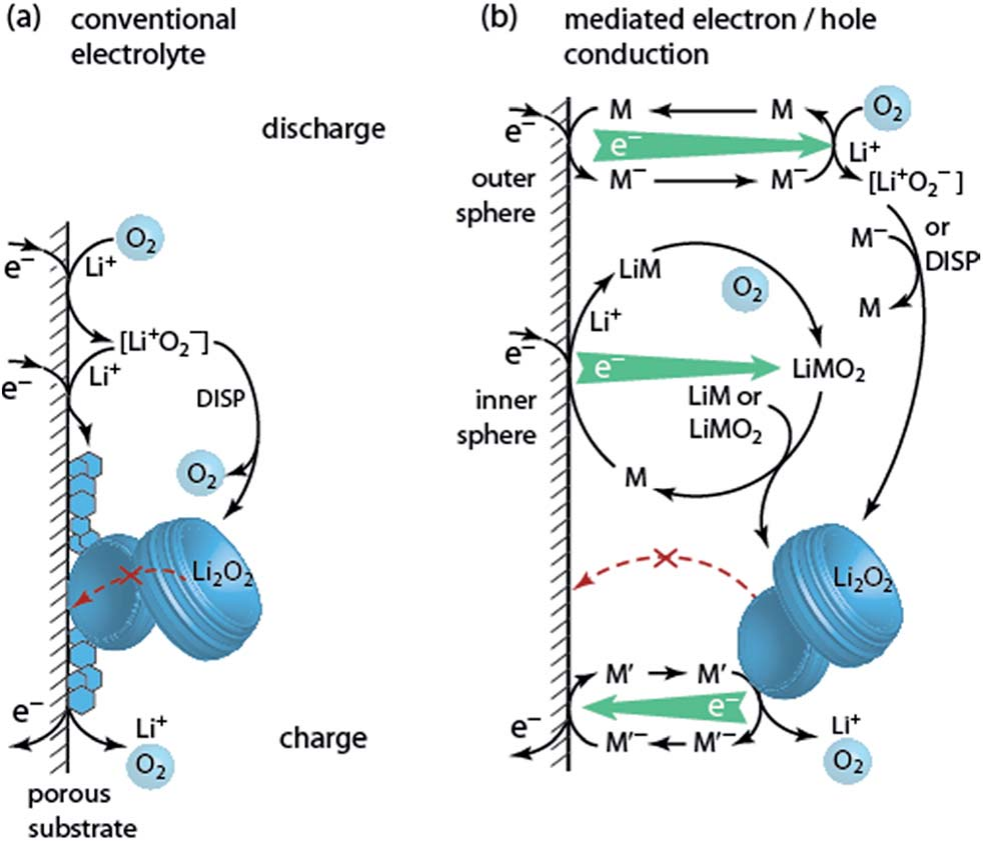
(a) Schematic of the reactions taking place in a Li–O_2_ cathode (O_2_ + 2Li^+^ + 2e^–^ ↔ Li_2_O_2_) during discharge and charge in conventional electrolyte. The insoluble and insulating discharge product Li_2_O_2_ forms on the surface of the conducting porous substrate and passivates it. Charging is hampered by poor electron transport. (b) Mediated electron/hole transport by mediators M and M′. The reduction mediator M may transfer electrons to O_2_ either in an outer sphere process or *via* an O_2_-binding transition state in an inner sphere process.

Lithium peroxide is a good charge storage medium with respect to formal capacity per mass and volume. It is, however, a poor medium with respect to the basic charge storage process of linking the redox moiety O_2_ to electron and ion transport according to O_2_ + 2e^–^ + 2Li^+^ ↔ Li_2_O_2_. However, different to other Li^+^ storage materials, Li^+^ and e^–^ transport into/out of the bulk Li_2_O_2_ particle is not even required since growth/dissolution occurs in any case at its surface. This unique feature of Li_2_O_2_ can be turned into a major advantage in terms of rate capability. It is the several orders of magnitude slower ion diffusivity compared to liquids that makes batteries slow and the very fast charge transport in liquids that makes supercapacitors high-power devices.^92^ Bypassing Li_2_O_2_ for ion and electron transport through a phase, where both are facile may thus enable high-power Li–O_2_ cells. Li_2_O_2_ would then only serve as the charge storage medium. While the liquid electrolyte (where the reaction takes place) provides facile ions transport, moving electrons through the liquid is more difficult. Possibilities involve: (a) giving solubility to LiO_2_ for it to act as an electron mediator during discharge (Fig. 7a, discussed in Section 3); (b) redox mediators that are reduced/oxidized at the e^–^ conductor, then move through the electrolyte and act in a distant position to reduce O_2_ or oxidize Li_2_O_2_, thereby being regenerated themselves (Fig. 7b).

Two classes of reduction mediators have been put forward. With the first, the reduced mediator M^–^ reduces O_2_ in an outer sphere reaction to superoxide, which then can undergo disproportionation or is further reduced by another M^–^ (Fig. 7b top), with investigated compounds including viologens and *N*-heterocyclic complexes.^93–95^ However, improvement of discharge capacity has not clearly been shown. Recently, particular quinones (*e.g.*, 2,5-di-*tert*-butyl-1,4-benzoquinone (DBBQ)) were shown to form Li_2_O_2_ in an inner sphere process without the involvement of free superoxide (Fig. 7b centre).^96^ Reduction of the quinone in presence of Li^+^ and O_2_ leads to a LiM and then LiMO_2_ complex. The latter is more stable than [Li^+^O_2_^–^] as seen by the higher discharge potential, and disproportionates to form Li_2_O_2_ and to reform M. LiMO_2_ dissolves even in poorly solvating glyme electrolytes, thus mitigating the trade-off between solubilisation and stability and allowing for substantially increased discharge capacity.^23,96^ Higher Li_2_O_2_ yield with DBBQ than without it was attributed to the absence of free superoxide, albeit it is still unclear whether the quinone suppresses the direct reactivity of the superoxide or ^1^O_2_ formation.^24^

Oxidation mediators allow, in principle, charging at nearly zero overpotential and numerous oxidation mediators have been explored for redox potential and O_2_ evolution efficiency (e^–^/O_2_).^18,97–101^ Early work has found that some oxidation mediators with suitable redox potentials oxidize Li_2_O_2_ with the expected amount of O_2_ evolution whereas others with similar potential evolve considerably less O_2_.^98^ As mechanistic descriptor for this behaviour, the position of the HOMO level of M^+^ was put forward, which, when close to the HOMO of the electrolyte, is prone to oxidize the electrolyte.^99^

Two pitfalls have to be considered with mediators: first, oxidation mediators M^+^ may, instead of oxidizing Li_2_O_2_, diffuse out of the cathode to the counter electrode to cause leak-current by shuttling. This may be avoided by using Li^+^ conducting diffusion barriers such as ceramics or polymers as the separator.^101^ Second, mediators, which are mostly organic molecules, are themselves susceptible to decomposition. Both issues make it imperative to quantitatively measure O_2_ consumption/evolution, Li_2_O_2_ formation/oxidation, and parasitic products as discussed in Section 4.1. Any claim about performance improvements is inadequate without these measures.

## 7. Outlook

The past few years have brought substantial progress with the mechanisms underpinning the operation of the Li–O_2_ cathode. The two central issues are: (A) discharge/charge mechanisms of Li_2_O_2_, and (B) mechanisms of parasitic chemistry.

A central issue was to identify conditions leading to discharge *via* a surface passivating mechanism (giving low capacities) or a solution based process to form large Li_2_O_2_ particles (required for high capacities). The deciding factor is the solvation of the superoxide intermediate by tuning the electrolyte interaction with Li^+^ or O_2_^–^ *via* solvent, salt, and additive Lewis basicity/acidity. Oxidation of Li_2_O_2_ proceeds at low kinetic overpotentials and can thus, in principle, take place at high rates close to the thermodynamic potential. Rising voltage is predominantly associated with parasitic chemistry. Nevertheless, when discharge proceeds *via* the solution mechanism charge transport from large Li_2_O_2_ particles will contribute to overpotentials.

Solution based Li–O_2_ chemistry appears to be the way forward for high capacity and rate capability and low overpotentials, here, Li_2_O_2_ only serves as the storage medium and is bypassed for charge transport through the electrolyte by means of redox mediators. With reduction mediators the pathway to form Li_2_O_2_ may be altered such that there is not free superoxide, which is a source of singlet oxygen and thus parasitic chemistry on discharge. Oxidation mediators allow, in principle, for charging at nearly zero overpotential. The biggest open question with mediators is their own susceptibility to decomposition and their impact on singlet oxygen formation.

The major barrier for reversible cell operation is parasitic chemistry with electrolyte and cell components. The previous view of superoxide and Li_2_O_2_ being the major cause was only recently overturned by finding that singlet oxygen (^1^O_2_) is formed on discharge and charge; the extent matches the pattern of parasitic reactions with relatively little on discharge and much more on charge. Practical realization of Li–O_2_ batteries will, in our opinion, stand or fall with mastering ^1^O_2_ formation. Open questions centre around: (1) factors influencing ^1^O_2_ formation including catalysts, electrolytes, mediators, and protic additives; (2) more detailed insight into formation mechanisms; (3) finding efficient quenchers; (4) finding mechanism to prevent ^1^O_2_ formation. Given that so far significant parasitic chemistry is to be expected during both discharge and charge, concluding about the efficacy of any measure to improve capacity, efficiency and cyclability requires quantitative analysis of reactant turnover and parasitic products without which any claim of improvement is inadequate.

There is no theoretical barrier for the Li–O_2_ cathode to achieve much higher capacity than intercalation cathodes. However, to do so it is crucial to achieve an as high as possible packing density of Li_2_O_2_ in the cathode and to minimize the inactive mass and volume including the electrolyte. Two habits make tracing progress in the field difficult: first, reporting capacity with respect to porous substrate mass, which represents a minor and widely varying fraction of the total electrode mass; second, reporting cycling at, *e.g.*, 1000 mA h g_carbon_^–1^, which may still seem a lot in comparison to intercalation electrodes. The problem is that in most cases true performance is below intercalation electrodes and that it masks irreversible reactions. Therefore, it is important to report performance with respect to the full electrode to allow for a fair assessment of energy, power, and cycle life.
